# The impact of iron limitation on the physiology of the Antarctic diatom *Chaetoceros simplex*

**DOI:** 10.1007/s00227-014-2392-z

**Published:** 2014-01-29

**Authors:** Katherina Petrou, Scarlett Trimborn, Björn Rost, Peter J. Ralph, Christel S. Hassler

**Affiliations:** 1 Plant Functional Biology and Climate Change Cluster, University of Technology, Sydney, Australia; 2 Alfred Wegener Institute for Polar and Marine Research, Am Handelshafen 12, 27570 Bremerhaven, Germany; 3 Institute FA Forel, University of Geneva, 10 rte de Suisse, 1290 Versoix, Switzerland

## Abstract

Iron availability strongly governs the growth of Southern Ocean phytoplankton. To investigate how iron limitation affects photosynthesis as well as the uptake of carbon and iron in the Antarctic diatom *Chaetoceros* *simplex*, a combination of chlorophyll *a* fluorescence measurements and radiotracer incubations in the presence and absence of chemical inhibitors was conducted. Iron limitation in *C. simplex* led to a decline in growth rates, photochemical efficiency and structural changes in photosystem II (PSII), including a reorganisation of photosynthetic units in PSII and an increase in size of the functional absorption cross section of PSII. Iron-limited cells further exhibited a reduced plastoquinone pool and decreased photosynthetic electron transport rate, while non-photochemical quenching and relative xanthophyll pigment content were strongly increased, suggesting a photoprotective response. Additionally, iron limitation resulted in a strong decline in carbon fixation and thus the particulate organic carbon quotas. Inhibitor studies demonstrated that, independent of the iron supply, carbon fixation was dependent on internal, but not on extracellular carbonic anhydrase activity. Orthovanadate more strongly inhibited iron uptake in iron-limited cells, indicating that P-type ATPase transporters are involved in iron uptake. The stronger reduction in iron uptake by ascorbate in iron-limited cells suggests that the re-oxidation of iron is required before it can be taken up and further supports the presence of a high-affinity iron transport pathway. The measured changes to photosystem architecture and shifts in carbon and iron uptake strategies in *C. simplex* as a result of iron limitation provide evidence for a complex interaction of these processes to balance the iron requirements for photosynthesis and carbon demand for sustained growth in iron-limited waters.

## Introduction

The Southern Ocean is the largest CO_2_ sink in the global ocean and therefore plays a key role in the global climate (Sabine et al. 2004). The biological carbon pump in this region is driven by autotrophic photosynthetic activity, yet it operates at sub-optimal levels, as the growth and activity of primary producers is limited by iron (Martin 1990). Due to iron limitation, large parts of the Southern Ocean are classified as high-nutrient, low-chlorophyll (HNLC) areas. Next to these HNLC areas, there are several regions of high primary productivity, reflected by the presence of large phytoplankton blooms. These blooms usually occur in naturally iron-enriched regions, such as the sea ice edge (Lannuzel et al. 2008), polynyas, continental margins (Lam et al. 2006) and ocean upwelling or circulation fronts (e.g., Polar Frontal Zone; de Baar et al. 1997). As these phytoplankton blooms develop, however, the overall consumption of iron increases, often to a level greater than the input of iron, causing iron limitation to occur, even in principally iron-enriched regions (Garibotti et al. 2005).

Iron is an essential micronutrient for phytoplankton, being involved in cellular processes such as photosynthesis, nitrate reduction, N_2_ fixation as well as providing protection from reactive oxygen species (Geider and La Roche 1994; Morel and Price 2003). Most of the cell's requirement (up to 80 %) is associated with photosynthesis (Raven 1990), where iron functions as an integral part of both photosystem I and II and various cytochromes of the photosynthetic electron transport chain (Greene et al. 1991, 1992). It is therefore not surprising that many studies have investigated the complex link between light and iron in oceanic systems (Boyd et al. 2001; Petrou et al. 2011; Alderkamp et al. 2012; Strzepek et al. 2012).

To minimize their iron requirements, open-ocean species have lower concentrations of photosystem I and cytochrome b_6_f (Strzepek and Harrison 2004), decrease their cellular pigment concentrations at the cost of light capture efficiency (Petrou et al. 2011) and/or substitute iron-containing enzymes such as ferredoxin with flavodoxin and proteins with iron-free equivalents (La Roche et al. 1996; Marchetti et al. 2009). Another strategy of phytoplankton under iron deficiency is to induce a high-affinity transport system to acquire iron (Maldonado and Price 1999; Maldonado et al. 2006). Eukaryotic phytoplankton such as diatoms mainly acquire iron by the reductive iron uptake pathway, involving two plasma membrane proteins (a reductase and a permease), as well as two iron redox transformations (Maldonado et al. 2006; Shaked and Lis 2012). Antarctic diatoms, as well as the flagellate *Phaeocystis antarctica,* are known to reduce and assimilate iron using strong organic ligands linked to reductases located on the cell surface (Strzepek et al. 2011).

Years of research have identified consistent changes in iron-limited phytoplankton photophysiology. Due to the central role of iron in both photosystems and the photosynthetic electron transport chain, iron limitation causes a major reorganization of the thylakoid architecture (Raven 1990). This reorganization causes a disconnection between light-harvesting centers resulting in a decline in photosystem II efficiency, electron transport and carbon fixation under iron-limited conditions (reviewed in Behrenfeld and Milligan 2012). Lowered electron transport rates can lead to reduced production of adenosine triphosphate (ATP) and nicotinamide adenine dinucleotide phosphate hydrogen (NADPH), energy equivalents that are needed to drive iron and carbon uptake as well as the reduction and assimilation of inorganic carbon. Carbon assimilation is an energy-requiring process for the cell, as CO_2_ needs to be actively concentrated at the catalytic site of the carboxylating enzyme ribulose-1,5-bisphosphate carboxylase/oxygenase (RubisCO) to compensate for its low affinity for CO_2_. The operation of such carbon-concentrating mechanisms involves the active uptake of CO_2_ and/or HCO_3_^−^ and the expression of varying activities of external and internal carbonic anhydrase, which accelerates the conversion between HCO_3_^−^ and CO_2_ (Reinfelder 2011). To take up inorganic carbon in an efficient manner may become especially important with increasing iron limitation, when energy availability gets more and more constrained. Consequently, there might be trade-offs between a reduced energy supply resulting from lower electron transport rates and the energy needed for inorganic carbon and iron acquisition under iron limitation (Raven 1990). Schulz et al. (2007) showed that iron limitation had a strong impact on carbon acquisition, reducing carbon uptake and fixation rates in a calcifying microalga. How these processes interact with iron acquisition in Southern Ocean phytoplankton is currently unknown.

In this study, we investigate the response of growth, photophysiology, carbon and iron uptake for the Antarctic diatom *Chaetoceros simplex* under iron limitation. This study uses a combination of chlorophyll *a* fluorescence measurements and radiotracer incubations in the presence and absence of chemical inhibitors to better understand the modes of iron and carbon acquisition in *C. simplex*. We hereby try to unravel the physiological trade-offs and nutrient acquisition strategies for diatoms in a late bloom scenario, when the bioavailability of iron has become exhausted.

## Materials and methods

### Cultures

Cultures of the Antarctic diatom *C. simplex* (CS 624, ANACC, 3–5 μm) were isolated from the coastal waters of Antarctica (Prydz Bay) and maintained at 4 °C in sterile-filtered (0.2 μm) Southern Ocean seawater (0.28 nmol L^−1^ Fe, Hassler et al. 2011). This strain is growing under iron-limited conditions since 2008 in our laboratory, and its response to iron limitation is known (Hassler and Schoemann 2009a). Three months prior to the experiment, cultures of *C. simplex* were transferred into experimental conditions and left to grow at 30 μmol photons m^−2^ s^−1^ in natural seawater collected from the sub-Antarctic region of the Tasman Sea (46.3°S 159.9°E, 25 m, 0.65 nmol L^−1^ Fe, during PINTS, January–February 2010, *RV Southern Surveyor*) following the trace metal clean sampling techniques described by Bowie and Lohan (2009) and amended with chelated macronutrients (N, P, Si) according to the Redfield ratio (30 : 1.9 : 30 μmol L^−1^, respectively). The light level was selected based on previous tests that showed maximum quantum yield of PSII (*F*_V_/*F*_M_) to be greatest when cells were grown at this irradiance under iron-replete conditions.

### Experimental setup

For the experiment, cultures were transferred into eight acid-washed 4-L clear polycarbonate bottles (Nalgene) and incubated at 4 °C. Treatments consisted of 4 of these bottles enriched with 2 nmol L^−1^ of FeCl_3_ (ICP-MS standard, Fluka; +Fe) and the remaining 4 bottles enriched with 10 nmol L^−1^ of desferroxamine B (Sigma; +DFB). FeCl_3_ was added without EDTA addition as inorganic solubility equals 0.5 nM at 5 °C (Liu and Millero 1999) and iron-binding organic ligands (L) were present in excess to buffer the additional Fe concentration (L = 3.4 nM L^−1^; Norman et al., unpublished data). Desferroxamine B binds to iron and was used to reduce iron bioavailability in this strain (Hassler and Schoemann 2009a; Hassler et al. 2011). Incubation time lasted between 7 and 15 days depending on experimental treatment (+DFB incubations lasted longer than +Fe treatments). The pH of the seawater was 8.49 ± 0.04 and 8.52 ± 0.04 for the +Fe and +DFB acclimations, respectively. The pH was measured on a daily basis using a pH–ion meter (WTW, model pMX 3000/pH, Weilheim, Germany). Light was supplied at 30 μmol photons m^−2^ s^−1^ (Grolux, GMT lighting, Northmead, Australia) on a 16:8 h light:dark cycle.

### Iron chemistry

Dissolved iron concentrations were measured at the beginning and end of the experiment for each treatment. Measurements were performed at the University of Tasmania (Australia) using flow injection (de Jong et al. 1998). The experimental growth media was filtered (0.2 μm, polycarbonate filter, Millipore) and acidified (0.2 % v:v, quartz distilled HCl, Seastar) under trace metal clean conditions before analysis. All water manipulation and sampling was conducted using established trace metal clean techniques (Bowie and Lohan 2009).

### Growth rates

Cell count samples were taken at the same time each day, immediately fixed with Lugol’s solution (1 % final concentration) and stored at 4 °C in the dark until counting. Cell numbers were estimated using Utermöhl chambers on an inverted microscope (Zeiss Axiovert 200). Each sample was examined at magnification of 400× until at least 400 cells had been counted. Cell-specific growth rate (μ unit day^−1^) was calculated as

1 $$\mu = \left( {\ln N_{\text{fin}} {-}\ln N_{0} } \right)/\varDelta t$$

where *N*_0_ and *N*_fin_ denote the cell concentrations at the beginning and the end of the experiments, respectively, and ∆*t* is the corresponding duration of incubation in days.

### Particulate organic carbon (POC)

Subsamples from each bottle were gently filtered (<20 mmHg) onto pre-combusted 25-mm GF/F filters (Whatman, USA) and stored at −80 °C. Prior to analysis, filters were defrosted, acidified with 0.1 N HCl and dried overnight at 60 °C. Particulate organic carbon (POC) was measured using an isotope ratio mass spectrometer (IRMS; Delta V, Thermo Finnigan). Carbon isotopes were derived by comparison with calibrated external standards introduced during analysis and reported against PEE DEE Belemnite (PBD). Final concentration of POC was normalized per cell.

### Pigments

Pigment concentrations were determined using high-performance liquid chromatography (HPLC). Samples were filtered onto 25-mm GF/F filters (Whatman), immediately frozen in liquid nitrogen and stored in the dark at −80 °C for later analysis. Pigments were extracted and analyzed according to the methods of van Heukelem and Thomas (2001) with the only modification being an extra filtration step through 0.2 μm PTFE 13-mm syringe filters (Micro-Analytix Pty Ltd.). Clarified samples were stored in amber HPLC glass vials (Waters Australia Pty Ltd., Woolloongabba, Australia) and were stored at −80 °C overnight before analysis. The HPLC system included a pump, temperature-controlled auto-injector (Waters Australia Pty Ltd., Woolloongabba, QLD, Australia), C8 column (150 × 4.6 mm; Eclipse XDB), and photodiode array detector (Waters Australia Pty Ltd., Woolloongabba, Australia). Pigments were identified by comparison of their retention times and spectra using calibration standards (DHI, Horsholm Denmark) for each pigment. Dilutions of the standard were injected into the HPLC for a five point calibration curve. Peak area was integrated using Empower Pro software (Waters Australia Pty Ltd., Woolloongabba, Australia) and checked manually to confirm the accuracy of the peak baselines and the similarity of the integrated peaks to that of the standard.

### Chlorophyll *a* fluorescence

To assess the photosynthetic status of cells under iron limitation, steady-state light curves were performed 4–8 h after the onset of light using a pulse-amplitude-modulated fluorometer (Water-PAM; Walz GmbH, Effeltrich, Germany). A 3-mL aliquot of the respective treatment was transferred to a quartz cuvette and dark-adapted for 10 min under continuous stirring, before minimum fluorescence (*F*_O_) was recorded. This dark-adaptation period was chosen after testing different time intervals (5, 10, 15 and 20 min) to ensure largest possible *F*_M_ prior to measurements. Upon application of a saturating pulse of light (pulse duration = 0.8 s; pulse intensity >3,000 μmol photons m^−2^ s^−1^), maximum fluorescence (*F*_M_) was determined. From these two parameters, maximum quantum yield of PSII (*F*_V_/*F*_M_) was calculated according to the equation (*F*_M_ − F_O_)/*F*_M_ (Schreiber 2004). During the steady-state light curve, nine actinic light levels (28, 42, 65, 100, 150, 320, 680, 1,220 and 2,260 μmol photons m^−2^ s^−1^) were applied for 5 min each before recording the F_O_’ (light-adapted minimum fluorescence) and *F*_M_′ (light-adapted maximum fluorescence) values. From these light-adapted fluorescence yields, effective quantum yield of PSII was calculated for each irradiance level according to the equation *F*_M_ − *F*_O_′)/*F*_M_′ (Schreiber 2004). After the completion of the light curve, an additional dark-adaptation period of 10 min was applied, followed by a saturating pulse, to check for recovery and/or any permanent damage to photosystem II. All measurements were conducted at growth temperature (4 °C). Non-photochemical quenching of chlorophyll *a* fluorescence (NPQ) was calculated as (*F*_M_ − *F*_M_′)/*F*_M_′. Relative electron transport rates (rETR) were calculated as the product of effective quantum yield (*Φ*_PSII_) and actinic irradiance. Electron transport as a function of irradiance was fitted, and all photosynthetic parameters including maximum rETR (rETR_MAX_), minimum saturating irradiance (*E*_K_) and maximum light utilization efficiency (α) were derived according to Ralph and Gademann (2005).

To assess the changes in different components of the electron transport chain, fast induction curves (FICs) were measured on filter-concentrated samples from each treatment, using a double-modulation fluorometer (Photon System Instruments, FL-3500, Brno, Czech Republic) with a 3-s multiple turnover flash at >3,000 μmol photons m^−2^ s^−1^ light intensity. The FIC goes through four transient steps from base fluorescence (*F*_O_) to maximum fluorescence (*F*_M_). These steps are commonly denoted as O, J, I and P, respectively (Strasser and Govindjee 1991; Strasser et al. 1995), where the O–J step involves the passing of the electron from PSII to the primary electron acceptor *Q*_A_, the J-I transient is linked with the reduction in the secondary quinone *Q*_B_ and finally the P step, which is reached once the PQ pool is filled, represents the re-oxidation of *Q*_A_^−^ (Strasser et al. 1995). Prior to measurements, tests were done to ensure that cells were not damaged due to filtration (as indicated by unaffected *F*_V_/*F*_M_). Fluorescence measurements on dark-adapted (10 min) samples were recorded every 10 μs for the first 2 ms, every 1 ms until 1 s, then every 500 ms up to 3 s. All FICs were normalized to *F*_O_, where all values were divided by the O step (at 50 μs). Effective absorption cross-sectional area (*σ*_PSII_), the ratio of PSII α and β centers (PSIIα:β), and PSII connectivity (Jcon) were calculated from a single turnover flash of 80 μs at >3,000 μmol photons m^−2^ s^−1^ light intensity in accordance with the methods outlined in Nedbal et al. (1999).

### Chemical inhibitor experiments

To better understand the role of iron in photosynthesis and gain greater insight into iron and carbon uptake physiology, a suite of specific inhibitors were used on the +Fe and +DFB cultures. A total of nine different inhibitors (Table 1) were assessed in terms of their effect on photosynthesis, carbon and iron uptake. Orthovanadate (Van, British Drug Houses Company), an inhibitor of the plasma membrane-bound P-type ATPases, was used to inhibit the active uptake of iron and carbon, while diuron (DCMU, Sigma) was used to inhibit linear electron transport. To investigate the form of iron taken up, ascorbate (Asc, Sigma) and ferrozine (Fer, Tokyo Chemical Industry Ltd.,) were applied individually and in combination. Methyl viologen (MV, Sigma) was used to prevent the reduction in the photosynthetic electron transport chain and maintain ATP production. To investigate the effects of iron limitation on carbon uptake, two inhibitors for carbonic anhydrase were applied: ethoxzolamide (EZA, Sigma), inhibits both internal and external carbonic anhydrase (CA), while acetazolamide (AZA, Sigma) blocks only external CA. Carbonyl cyanide *m*-chlorophenyl hydrazone (CCCP, Sigma) was used to inhibit oxidative phosphorylation. Concentration for each inhibitor (Table 1) was determined based on a series of titrations. The chosen concentration for each inhibitor was the lowest at which a photophysiological effect was evident.

**Table 1:** List of inhibitors used, their final concentrations and their biological and chemical modes of action

Inhibitor	Final concentration (μmol L^−1^)	Biological and chemical mode of action
Van	50 (0.1–50)	Inhibits ATP use for transport by P-type ATPase^a^
DCMU	0.5 (0.1–50)	Inhibits photosynthetic electron transport^b^
Asc	1,000 (100–10,000)	Reduces Fe(III)–Fe(II)^c^
Fer	100 (1–100)	Complexes Fe(II)–Fe(III)^d^
MV	100 (0.5–100)	Strong electron acceptor, maintains active electron transport^b^
EZA	500 (50–1,000)	Inhibits extra- and intracellular carbonic anhydrase^e^
AZA	100 (10–200)	Inhibits extracellular carbonic anhydrase^e^
CCCP	10 (0.5–100)	Inhibits oxidative phosphorylation^f^

The final concentration of inhibitor used in our experiments was defined as the concentration causing approx. 50 % of inhibition of maximum quantum yield of PSII and rETR applying different concentrations of each inhibitor (concentration range tested shown in brackets). When no decrease in the maximum quantum yield of PSII was observed, the concentration was set to its maximum or to a concentration previously reported effective on phytoplankton. Inhibitors were orthovanadate (Van), diuron (DCMU), ascorbate (Asc), ferrozine (Fer), methyl viologen (MV), ethoxzolamide (EZA), acetazolamide (AZA) and carbonyl cyanide *m*-chlorophenyl hydrazone (CCCP)

^a^Meisch and Becker (1981), ^b^ Duysens (1979), ^c^ Maldonado and Price (2001), ^d^ Shaked et al. (2005), ^e^ Palmqvist et al. (1994), ^f ^Raven and Glidewell (1975)

Stock solutions for each inhibitor were freshly prepared, stored according to supplier guidelines and used within a period of 3 weeks. Four cycles of 10-min heating (80 °C) and cooling (4 °C) with pH adjustment between each cycle were used to ensure that the stock solution of Van (10 mmol L^−1^ in MilliQ water, Sartorius, <18 MΩ, pH = 7.2) was in its orthovanadate form as suggested by Furla et al. (2000). The stock solutions of Asc (10 mmol L^−1^) and Fer (10 mmol L^−1^) were prepared in MilliQ water. The stock solutions of DCMU (1 mmol L^−1^) and CCCP (2.4 mmol L^−1^) were prepared in ethanol (Sigma, ACS grade), whereas MV was prepared in 1:1 ethanol: MilliQ water, resulting in ethanol addition of 0.05, 0.42 and 0.64 % in the experimental media, respectively. The stock solution of EZA (50 mmol L^−1^) and AZA (20 mmol L^−1^) were prepared in DMSO and 1:1 DMSO: MilliQ water, respectively, resulting in a DMSO addition of <1 % in the experimental media. The effect of ethanol and DMSO addition on the parameters measured was quantified as a procedural blank in duplicate and used to correct the results from the inhibitor experiments for CCCP, MV, EZA and AZA.

Before fluorometric measurements were made, volumes (30 mL) of +Fe and +DFB cultures were inoculated with the respective concentration of chemical inhibitor (Table 1) for 24 h under growth conditions. Chlorophyll *a* fluorescence measurements of inhibitor-exposed cells were performed 4 h after the onset of light using the same Water-PAM as described above. A 3-mL aliquot of sample was transferred into a quartz cuvette and left in the dark for 10 min before maximum quantum yield was determined.

### Bioaccumulation experiments with ^14^C and ^55^Fe

Iron and carbon uptake was measured following a 24-h incubation (4 °C with 30 μmol photons m^−2^ s^−1^ light intensity) using radioisotopes (Perkin Elmer, 20.82 mCi mg^−1^ Fe at the time of experiment and ^14^C 1 mCi mL^−1^) in the presence and absence of chemical inhibitors (described above for chlorophyll *a* fluorescence measurements and in Table 1). Bioaccumulation experiments and calculations were carried out as in Hassler and Schoemann (2009a). Solution for the uptake experiments consisted of artificial seawater (major salts, macronutrients only, Wake et al. 2012) with 90 nmol L^−1^ of total Fe and 110 nmol L^−1^ EDTA (Sigma). The iron concentrations present in the inhibitor (detected by Inductive Coupled Plasma Mass Spectroscopy by the team of Dr. E. Butler at CSIRO, Hobart, Australia) and the radioactive iron added were considered in the total iron concentration of the solutions. Bioaccumulation solution with radioisotopes (10 nmol L^−1^ ^55^Fe and 5 μCi mL^−1^ ^14^C) and inhibitors were prepared 16–20 h in advance, stored in the dark at 4 °C to reach equilibrium and sampled for initial radioactivity (usually 1 mL) prior to bioaccumulation experiment. *C. simplex* was concentrated by gentle filtration (2 μm, polycarbonate filter, Millipore), rinsed in artificial seawater and re-suspended in the bioaccumulation solution.

At the end of the accumulation period (24 h), cells were collected by filtration (0.45 μm, nitrocellulose filter, Millipore), rinsed with filtered seawater (for ^14^C uptake, to reduce the background of ^14^C-DIC) or rinsed with oxalate solution and then filtered seawater (for ^55^Fe uptake, to remove extracellular ^55^Fe, Hassler and Schoemann 2009b). Each filter was collected in a scintillation vial and amended with scintillation cocktail (10 mL, Ultima Gold, Perkin Elmer) directly (^55^Fe uptake) or following a degassing phase (addition of 200 μL of HCl 2 mol L^−1^ to let the ^14^C-DIC degas overnight). Counts per minute were analyzed using a scintillation counter (Perkin Elmer TriCarb 2000), and uptake was expressed as percentage of the uptake in the control (no inhibitor). Bioaccumulation experiments were performed in triplicate for each of the isotopes (^55^Fe and ^14^C).

Because solvents used to prepare some inhibitors could affect the permeability of the biological membrane (e.g., Wang et al. 2011) and thus alter biological uptake of iron or carbon, their effect was measured to consider possible associated artefacts. For that purpose, ^55^Fe and ^14^C uptake was measured in the presence of identical DMSO and ethanol proportions. Ethanol and DMSO had no statistical effect on ^14^C uptake, but there was an increase in iron uptake by a factor of 2.0 ± 0.4 for DMSO (*n* = 5) and 1.9 ± 0.5 for ethanol (*n* = 5).

### Data analysis

Differences in physiological responses of cells between +Fe and +DFB treatments were assessed using a one-way analysis of variance (ANOVA) or standard *t* test (*α* = 0.05). To ensure that the assumption of equal variances for all parametric tests was satisfied, a Levene’s test for homogeneity of variance was applied to all data a priori. In the case of the pigment data where the +DFB treatment only had two replicates due to biomass limitations, a power analysis was performed on the ANOVA to check for type II error probability. All analyses were performed using Minitab statistical software (version 15.1.0.0 2006, PA, USA).

## Results

### Iron concentration, growth rate, carbon quota and pigments

Initial iron concentrations (measured in the media prior to the experiment) were 2.27 and 0.48 nmol L^−1^ for the +Fe and +DFB treatments, respectively. Final iron concentrations (measure at the end of the experiment when cells were harvested) were 1.12 ± 0.09 and 0.37 ± 0.06 nmol L^−1^ for the +Fe and +DFB treatments, respectively. Growth rates were significantly lower in the +DFB culture than in the +Fe culture (*P* > 0.001), with rates of 0.13 ± 0.07 and 0.33 ± 0.12 days^−1^, respectively (Table 2). Cellular particulate organic carbon (POC) was significantly lower in the +DFB treatment (*P* = 0.019; Table 2). No differences in light-harvesting pigment (Chl *a*, fucoxanthin) concentrations between the +Fe and +DFB treatments were detected (Table 2). However, the relative proportion of the epoxidized xanthophyll pigment, diadinoxanthin, was significantly greater in the +DFB cultures (*P* < 0.002).

**Table 2:** Growth rate (day^−1^), particulate organic carbon (POC; pg cell^−1^) and chlorophyll *a* quota (Chl *a*; pg cell^−1^) as well as pigment concentrations (μg g^−1^ Chl *a*) of +Fe and +DFB cultures of *C. simplex*

Treatment	Growth rate (day^−1^)	POC (pg cell^−1^)	Chlorophyll *a* (pg cell^−1^)	Fucoxanthin (μg g^−1^ Chl *a*)	Diadinoxanthin (μg g^−1^ Chl *a*)
+Fe	0.33 ± 0.12	9.01 ± 0.75	63.1 ± 8.0	0.445 ± 0.025	0.135 ± 0.023
+DFB	0.13 ± 0.07	5.89 ± 1.67	54.3 ± 21.1	0.457 ± 0.018	0.222 ± 0.012
ANOVA	*P* = 0.050	*P* = 0.019	ns* ^,*^*^	ns* ^,†^	*P* = 0.002^^^

Data represent mean ± SD (*n* = 4, +Fe; *n* = 3, +DFB), in case of pigment data (*n* = 4, +Fe; *n* = 2, +DFB). Statistical results are from a one-way ANOVA between treatments at significance *α* < 0.05

* *P* > 0.05; ^^^ power = 1.0; ^†^ power = 0.54

### Chlorophyll *a* fluorescence

**Fig. 1:**
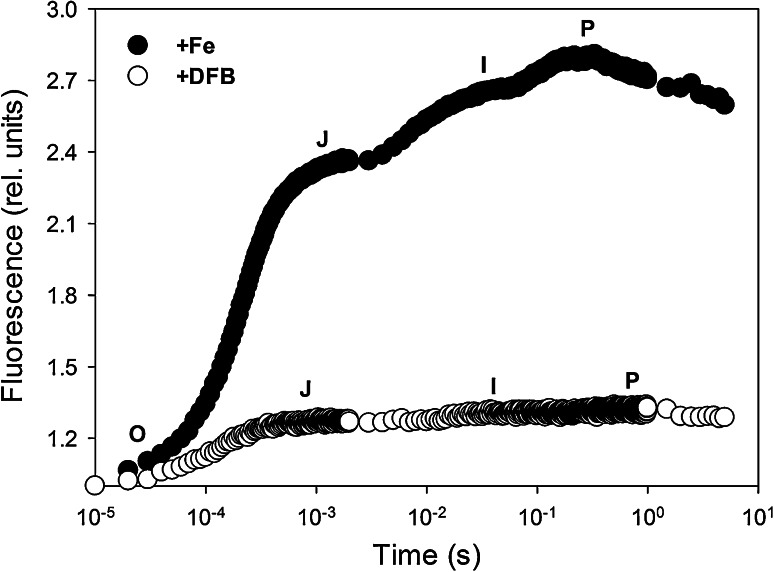
Fast induction curves (FICs) for +Fe (*filled circles*) and +DFB (*open circles*) cultures. Individual O–J–I–P steps of the FIC are denoted. *Curves* are plotted on a semi-log scale and represent the mean of independent curves (*n* = 4)

Chlorophyll *a* fluorescence showed clear differences in photosynthetic activity between the +Fe and +DFB cultures. Maximum quantum yield of PSII (*F*_V_/*F*_M_) was significantly lower in the +DFB cultures (*P* < 0.001), <50 % of that measured in the iron-enriched cells (Table 3). Under iron limitation, there was a significant increase in *σ*_PSII_ (*P* < 0.001), as well as a decline in the proportion of PSII α:β centers (*P* < 0.001) compared to the +Fe treatment (Table 3). Furthermore, there was a significant decrease in reaction center connectivity from 0.658 in +Fe cells to zero in the +DFB treatment (Table 3). FICs showed clear differences in both shape and amplitude, with a less acute slope of the initial rise and a flattening of the fluorescence rise in the +DFB-treated cells (Fig. 1). The amplitude of all FIC steps (J, I and P) was significantly higher in +Fe culture (*P* < 0.001), with more than a 50 % drop in amplitude in the +DFB cultures (Table 3).

**Table 3:** Photophysiological parameters from steady-state light curves and fast induction curves for iron-enriched (+Fe) and iron-limited (+DFB) *C. simplex* including: maximum quantum yield of PSII (*F*_V_/*F*_M_), recovered *F*_V_/*F*_M_ (r*F*_V_/*F*_M_) and recovered non-photochemical quenching (rNPQ) measured after 10-min dark adaptation following the steady-state light curve, effective absorption cross-sectional area (*σ*_PSII_), proportion of PSII α and β centers (PSIIα:β), photosystem II connectivity (Jcon), J, I and P, derived from OJIP fast induction curves, maximum relative electron transport rate (rETR_max_), minimum saturating irradiance (*E*_k_) and light utilization efficiency (*α*)

	+Fe	+DFB	ANOVA
*F*_V_/*F*_M_	0.609 ± 0.011	0.300 ± 0.035	*P* < 0.001
r*F*_V_/*F*_M_	0.575 ± 0.019	0.300 ± 0.020	ns
rNPQ	0.175 ± 0.046	1.844 ± 0.151	*P* < 0.001
*σ*_PSII_ (rel. units)	1.76 ± 3.95	3.65 ± 2.16	*P* < 0.001
PSIIα:β	2.423 ± 0.514	1.390 ± 0.367	*P* < 0.001
Jcon (rel. units)	0.658 ± 0.110	0.000 ± 0.000	*P* < 0.001
*J* (rel. units)	2.33 ± 0.07	1.27 ± 0.05	*P* < 0.001
*I* (rel. units)	2.63 ± 0.10	1.29 ± 0.05	*P* < 0.001
*P* (rel. units)	2.78 ± 0.14	1.31 ± 0.05	*P* < 0.001
rETR_max_ (μmol electrons m^−2^ s^−1^)	111 ± 4.97	42.6 ± 3.54	*P* < 0.001
*E*_k_ (μmol photons m^−2^ s^−1^)	202 ± 18.5	164 ± 33.2	*P* < 0.001
*α*	0.55 ± 0.03	0.26 ± 0.03	*P* < 0.001

Data represent the mean ± SD (*n* = 4, +Fe; *n* = 3, +DFB). Statistical results are from a one-way ANOVA at significance *α* < 0.05. ANOVA results are for tests between +Fe and +DFB treatments in all cases except r*F*_V_/*F*_M_, where data were tested for significant differences against the initial *F*_V_/*F*_M_ and rNPQ, which was tested against the NPQ measured at the highest irradiance (2,260 μmol photons m^−2^ s^−1^)

**Fig. 2:**
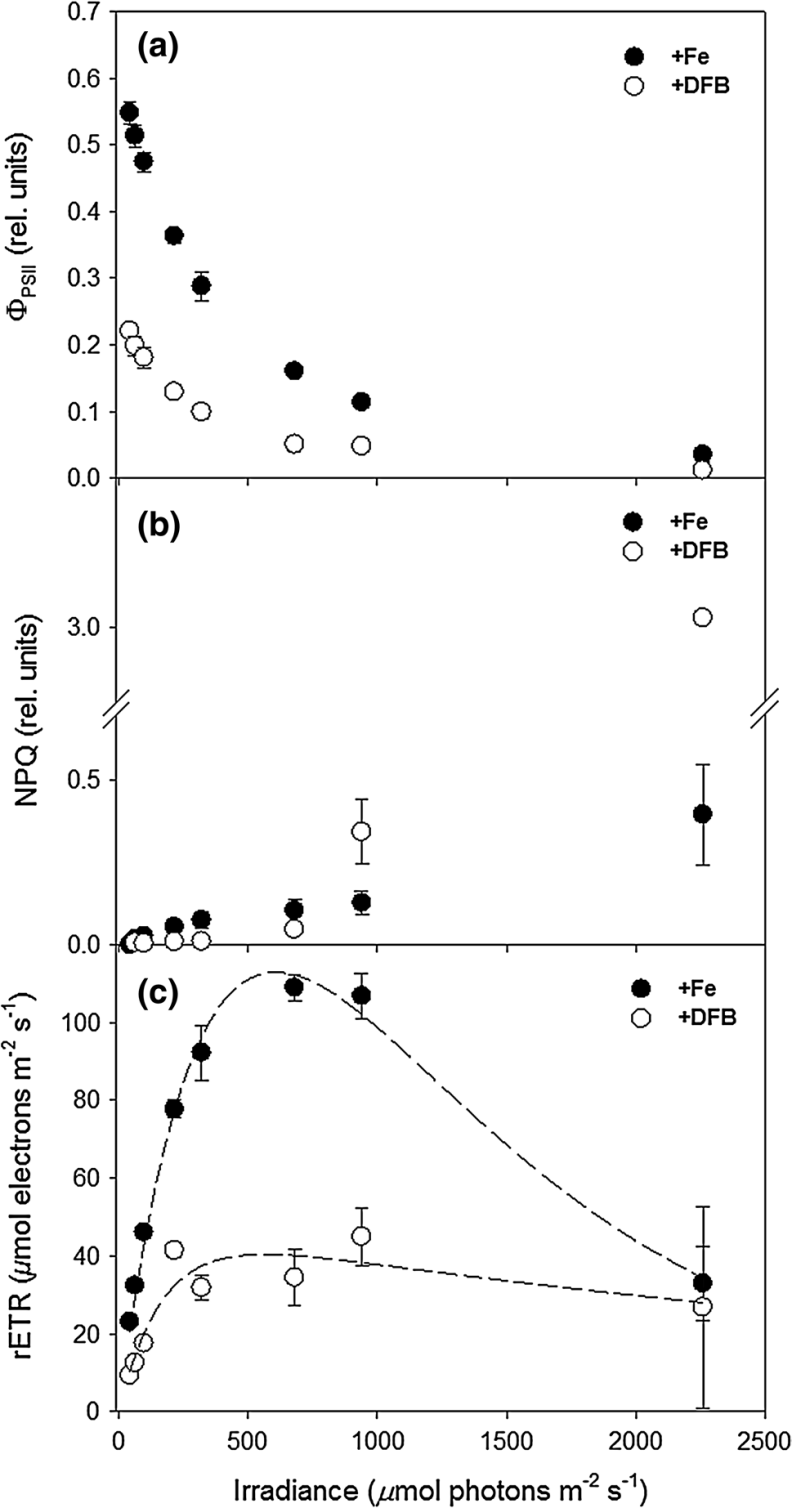
Fluorescence parameters. **a** Effective quantum yield of PSII ($\phi_{{_{\text{PSII}} }}$). **b** Non-photochemical quenching and **c** relative electron transport rate in +Fe (*filled circles*) and +DFB (*open circles*) cultures as a function of irradiance derived from steady-state *light* *curves*. Data represent mean ± SD (*n* = 4)

Steady-state light curves showed clear differences in *Φ*_PSII_ with lower values at all irradiance levels in the +DFB cultures (Fig. 2a). Initial *F*_V_/*F*_M_ values were <0.3 in the +DFB cultures compared with values of nearly 0.6 in the +Fe treatment (Fig. 2a). At the maximum irradiance (2,260 μmol photons m^−2^ s^−1^), however, yield values dropped well below 0.1 in both treatments (Fig. 2a). There was a significant increase in NPQ of the +DFB cultures at the highest irradiances (*P* < 0.001 at 1,220 and 2,260 μmol photons m^−2^ s^−1^), with NPQ values being twice as high as in +Fe cultures (Fig. 2b). There was complete recovery of *F*_V_/*F*_M_ (r*F*_V_/*F*_M_) following the additional 10-min dark-adaptation period applied at the end of the light curve with values returning to those measured initially for the +Fe cultures and the +DFB (Table 3). Additionally, after 10 min of darkness, rNPQ had declined significantly from 0.396 to 0.175 in the +Fe cultures (*P* < 0.001) and from 3.062 to 1.844 in the +DFB cultures (*P* < 0.001; Table 3). rETR was greater in the +Fe cultures than those measured in the +DFB cultures (Fig. 2c), showing yet similarly low values at the highest irradiance. In the +Fe cultures, there was evidence of photoinhibition only at the highest irradiance as indicated by a large drop in rETR, whereas rETR values remained consistently low in the +DFB cultures over the complete range of tested irradiances (Fig. 2c). The light curve parameters derived from Fig. 2c, rETR_max_, *E*_k_ and *α*, were all significantly lower in the +DFB cultures (*P* < 0.001; Table 3; Fig. 2c).

### Effect of inhibitors on chlorophyll *a* fluorescence

**Fig. 3:**
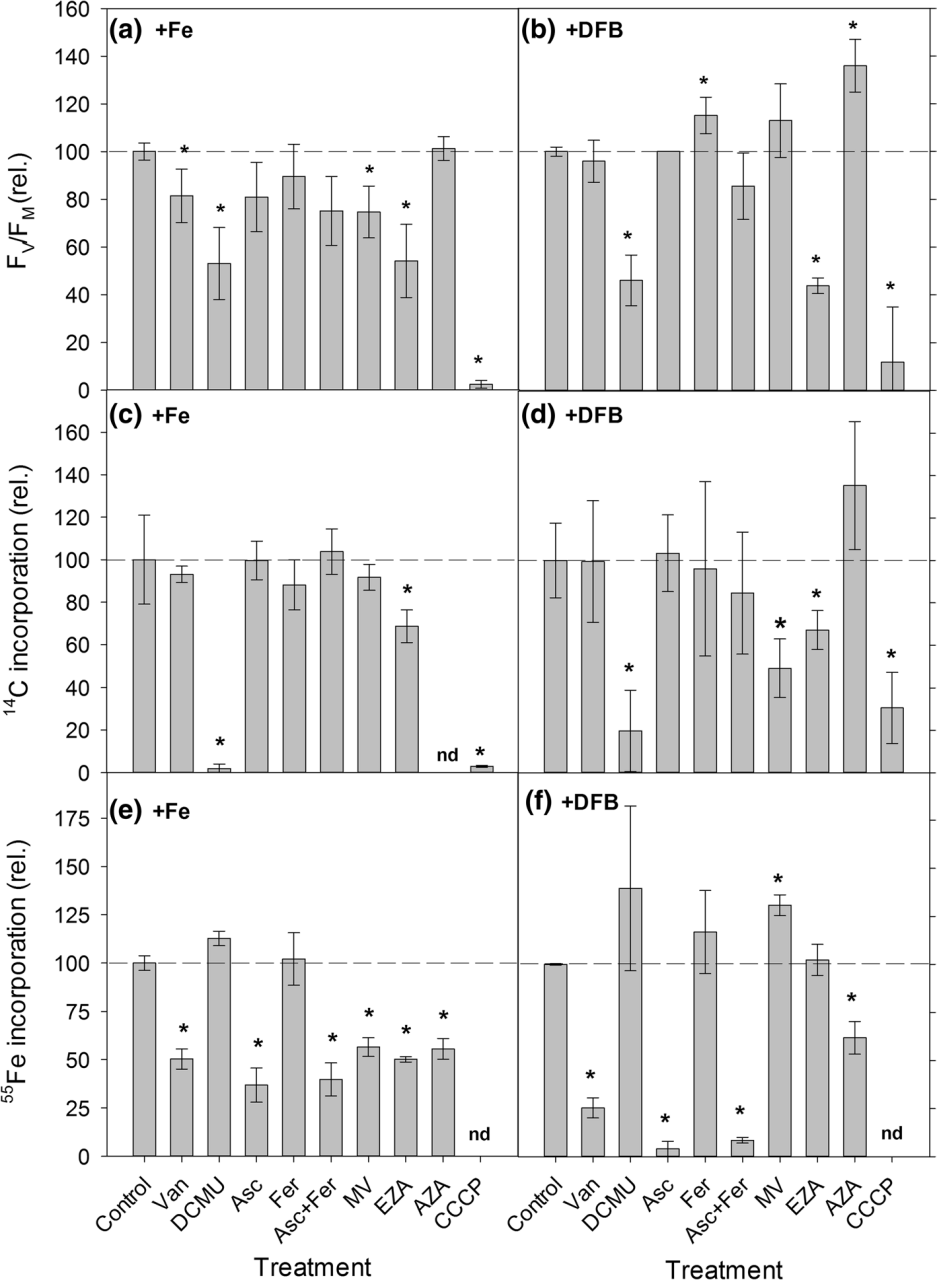
Maximum quantum efficiency of PSII (*F*_V_/F_M_) in **a** +Fe and **b** +DFB cultures treated with different inhibitors. Intracellular ^14^C incorporation in **c** +Fe and **d** +DFB cultures and intracellular ^55^Fe incorporation in **e** +Fe and **f** +DFB cultures in the presence of chemical inhibitors. Data are normalized to the control (*dashed horizontal line*) and represent the mean ± SD (*n* = 4, *F*_V_/*F*_M_; *n* = 3, ^14^C and ^55^Fe). *Data that are significantly different from the control at *α* < 0.05, *nd* represents no data

Maximum quantum yield of PSII varied in response to the different chemical inhibitors and between the +Fe and +DFB cultures (Fig. 3a, b). The greatest reduction in *F*_V_/*F*_M_ was in response to the photosynthetic electron transport inhibitor DCMU, the carbonic anhydrase inhibitor EZA and the inhibitor of oxidative phosphorylation CCCP in both the +Fe and +DFB treatments (Fig. 3a, b), with the effect of EZA being stronger in the +DFB culture. The addition of the P-type ATPase inhibitor Van and the strong electron acceptor MV resulted in a significant decline in *F*_V_/*F*_M_ in the +Fe cultures (*P* < 0.05), contrasting strongly to the lack of response in the +DFB cultures (Fig. 3a, b). Irrespective of the treatment, the addition of the inhibitor Asc, which reduces Fe(III)–Fe(II), did not affect *F*_V_/*F*_M_. Ferrozine, which complexes Fe(II)–Fe(III), elicited a positive response in the +DFB cultures, with an increase in *F*_V_/*F*_M_ of ~15 % compared to the control. Similarly, the external carbonic anhydrase inhibitor AZA resulted in a significant positive response in the +DFB cultures (40 % increase in *F*_V_/*F*_M_; *P* < 0.001; Fig. 3b).

### Effect of inhibitors on C and Fe uptake

In control treatments, carbon uptake decreased by sixfold in iron-limited compared to iron-enriched cells. In the presence of DCMU, EZA and CCCP, there was a decline in ^14^C incorporation in both the +Fe and +DFB cultures (*P* < 0.05; Fig. 3c, d). In comparison, CCCP had greater impact in the +Fe in comparison with +DFB treatments. The addition of MV resulted only in a decline in the +DFB treatments. AZA was only applied in +DFB treatments and induced an increased ^14^C incorporation with respect to the control (Fig. 3d). Under iron limitation, in absence of inhibitors, the iron uptake rates increased by a factor of two compared with cells grown under iron-replete conditions. Iron uptake was reduced significantly in the presence of Van, Asc, Asc + Fer, MV, EZA and AZA in the +Fe cultures (*P* < 0.05; Fig. 3e). For the +DFB cultures, iron uptake was significantly reduced by Van, Asc, Asc + Fer and AZA (*P* < 0.05; Fig. 3f). The effect of Van, Asc and Asc + Fer on iron uptake was significantly greater in +DFB treatment than in the +Fe cultures (*P* < 0.05; Fig. 3e, f). There was an opposite response for EZA and MV in the +DFB cultures to that seen in the +Fe cells, where ^55^Fe uptake in the iron-limited cultures was enhanced in the presence of these two inhibitors (Fig. 3f).

## Discussion

### Physiological responses to iron limitation

Iron limitation led to a 65 % decline in growth rate in *C. simplex* cells compared to those grown under replete conditions (Table 2). Reduced growth rates are commonly observed in Fe-limited phytoplankton (Timmermans et al. 2001; Alderkamp et al. 2012; Strzepek et al. 2012). In line with this, POC quotas were also reduced in +DFB cultures (Table 2), which suggests lower carbon fixation capacities. Along with these changes, several aspects of photophysiology were impacted by the differences in iron availability in *C. simplex*. In the +DFB-treated cells, for instance, an increased disconnection of antennae from PSII reaction centers is supported by our data, as Jcon was strongly reduced (Table 3). Congruently, also a shift from α-dominant PSII to a β-dominant PSII structure was observed under these conditions (Table 3), which indicates reorganization of the light-harvesting antenna systems into more isolated units (Lavergne and Briantais 1996). Consequently, the transfer of excitons to the PSII reaction centers is hindered, and thus, the efficiency of PSII is reduced, causing a decline in the *F*_V_/*F*_M_ in *C. simplex* grown in the +DFB medium (Table 3). This finding is consistent with general photosynthetic responses to iron limitation in phytoplankton (Greene et al. 1991, 1992; Vassiliev et al. 1994). Reduced *F*_V_/*F*_M_ in +DFB-treated cells of *C. simplex* was countered by an increase in σ_PSII_ (Table 3). Similar responses in σ_PSII_ were also observed for various iron-limited Southern Ocean diatoms (Timmermans et al. 2001; Van Oijen et al. 2004; Alderkamp et al. 2012; Strzepek et al. 2012). An increase in σ_PSII_ corresponds to an increase in the ratio of antenna complexes relative to the reaction center core complexes (Greene et al. 1992). Strzepek et al. (2012) suggested that a larger size of σ_PSII_ compensates for fewer iron-rich photosynthetic reaction centers in Southern Ocean phytoplankton species.

Iron is required in both photosystems (2–3 atoms for PSII; 12 atoms for PSI): the cytochrome *b*_*6*_ *f* complex (5 atoms), which links the two photosystems, and the ferredoxin molecule (2 atoms; Raven 1990). Given the substantial requirement of iron within the photosynthetic electron transport chain, Fe-limitation strongly influences electron transport kinetics (Fig. 2; Table 3). In line with a previous study (Beer et al. 2011), the significant drop in amplitude and flattening of the OJIP curves suggest slowing of electron transport and that the plastoquinone (PQ) pool was in a reduced state under iron limitation (Fig. 1). The reason for this is that re-oxidation of the PQ pool is dependent on the Cyt *b*_6_ *f* complex, which is impacted by iron limitation (Greene et al. 1992). The lack of iron inhibits the synthesis of functional components of the Cyt *b*_6_ *f* complexes (Bruce and Malkin 1991), thus resulting in the full reduction of the PQ pool and hindering electron transfer from PSII to PSI. As a result of this bottleneck, there is a buildup of protons and consequently an increase in heat dissipation in the form of NPQ (Fig. 2b). The complete recovery of *F*_V_/*F*_M_ and rapid relaxation of NPQ in the +DFB culture following the light curve would indicate that the majority of the high quenching observed was photoprotective and not photoinhibitory (Table 3). Correspondingly, the relative increase in diadinoxanthin content in the +DFB cultures (Table 2) suggests an increased investment into photoprotection, a response that has been previously recorded for iron-limited phytoplankton communities when exposed to high light (Petrou et al. 2011; Alderkamp et al. 2012, 2013).

### Effect of inhibitors on maximum quantum yield of PSII and carbon fixation

In *C. simplex*, the addition of Van, which blocks the plasma membrane-bound P-type ATPases (Gilmour et al. 1985; Palmqvist et al. 1988), had no inhibitory effect on ^14^C incorporation in +Fe and +DFB cultures (Fig. 3c, d), indicating that no P-type ATPase-dependent inorganic carbon uptake system was needed in this species. Orthovanadate can also inhibit the activity of phosphatases (Gallagher and Leonard 1982), an important group of enzymes involved in post-translational modification of proteins. Hence, our results also suggest a minor role of phosphatases in metabolic processes. In agreement with this, the temperate diatom *Chaetoceros muelleri* was also found to be insensitive to orthovanadate (Ihnken et al. 2011). Orthovanadate did, however, cause a decline in *F*_V_/*F*_M_, but only in the +Fe treatment (Fig. 3a, b), suggesting a greater demand on P-ATPases of the thylakoid membrane when electron transport rates are high and not compromised by iron limitation. Under iron-replete conditions, when ATPase activity is inhibited, the protons being generated through photosynthetic electron transport are not utilized by the ATPase, thus leading to proton buildup and consequently higher NPQ. In contrast, the lack of response to Van in the +DFB culture is likely due the already lowered ETRs and thus reduced proton gradient. Under these conditions, blocking ATPase by orthovanadate has no further effect.

As expected, the electron transport inhibitor DCMU (Duysens 1979) resulted in a decline in *F*_V_/*F*_M_ in *C. simplex* (Fig. 3a, b). DCMU blocks the Q_B_ binding site of PSII and therewith the electron transport between PSII and PSI (Ralph et al. 2010), meaning that less energy (NADPH and ATP) is available for inorganic carbon uptake and fixation. In agreement with this and previous studies (Sültemeyer et al. 1991; Bhatti et al. 2002), the addition of DCMU caused a pronounced inhibition in ^14^C incorporation in both +Fe and +DFB treatments (Fig. 3c, d). Similarly, the complete loss of *F*_V_/*F*_M_ by the inhibitor CCCP was expected (Fig. 3a, b), as it dissociates electron transport from ATP synthesis and impedes the establishment of a pH gradient across the thylakoid membrane (Ralph et al. 2010). Consequently, CCCP also strongly inhibited ^14^C incorporation in +Fe and +DFB cells (Fig. 3c, d).

Given that the inhibitors Asc and Fer were mainly used to identify the iron uptake strategy by *C. simplex,* it was not surprising that Asc had no effect on *F*_V_/*F*_M_ (Fig. 3a, b). Ferrozine, however, did result in an increase in *F*_V_/*F*_M_ in the +DFB cells, possibly as a result of some iron contamination. While care was taken to minimize iron input through the addition of 100 nM Fe buffered with 120 nM EDTA, Fer might have introduced additional iron into the solution.

Methyl viologen interacts at the binding site of ferredoxin on PSI, competing for the terminal electron and thus preventing the reduction of ferredoxin and the continued pathway of electrons to carbon fixation (Dan Hess 2000; Ralph et al. 2010). The addition of MV to *C. simplex* had no effect on *F*_V_/*F*_M_ in the +DFB cultures, but did cause a significant decline in the +Fe cultures (Fig. 3a, b). The reason for the different response in the +Fe and +DFB treatments is likely to be that MV can react with oxygen and produce the superoxide radical O_2_^−^ (Kohen and Chevion 1985). In presence of iron, O_2_^−^ can reduce Fe and react with hydrogen peroxide to produce the very reactive and deleterious hydroxyl radical (Zer et al. 1994), causing thus the decrease in *F*_V_/*F*_M_. Opposingly, DFB is known to chelate Fe, but also to scavenge free radicals; therefore, the effect by MV could have been reversed. According to our results, MV resulted in a strong inhibition of ^14^C incorporation in the +DFB treatment (Fig. 3c, d). This response is likely due to lowered electron transport under iron limitation, whereby all of the electrons being generated at PS II were effectively scavenged by the MV at PSI and therefore not utilized in carbon fixation.

Carbonic anhydrases (CAs) play an important role in inorganic carbon acquisition by accelerating the otherwise slow interconversion between HCO_3_^−^ and CO_2_. Next to various internal CAs, which all diatoms possess (Reinfelder 2011), most diatoms also have the potential to express high activities of external CA (e.g., Hopkinson et al. 2013; Trimborn et al. 2013). They can be selectively blocked by EZA and AZA. While AZA blocks CAs that are located at the cell surface, EZA additionally inhibits CAs inside the cell (Palmqvist et al. 1988). Independent of the Fe treatment, the presence of AZA did not alter ^14^C incorporation (Fig. 3c, d), indicating that *C. simplex* does not possess any extracellular CA and/or does not require it for carbon fixation under the applied conditions. In contrast, the addition of EZA led to a decline in ^14^C incorporation in both the +Fe and +DFB treatments (Fig. 3c, d), demonstrating the involvement of internal CAs in carbon fixation. A similar response was detected for photosynthetic efficiency, where EZA leads to a strong decline in *F*_V_/*F*_M_ in both treatments (Fig. 3a, b), lending support to *C. simplex’s* reliance on internal CAs to fuel carbon fixation in the Calvin cycle, the major sink for electrons from the photosynthetic electron transport chain.

### Effect of inhibitors on iron uptake

The dependence of the level of iron stress on iron uptake is well described, where cells in a low-iron environment can increase the density of iron transporters on the cell surface and induce high-affinity transporters (see Shaked and Lis 2012; Hassler et al. 2012 for recent reviews). Numerous studies have demonstrated that diatoms rely on a ferric reductase pathway to acquire iron (e.g., Maldonado and Price 2000; Shaked et al. 2005; Maldonado et al. 2006). Furthermore, genomic sequencing has indicated additional iron uptake pathways in *Thalassiosira pseudonana* (Kustka et al. 2007) and a putative ATP-binding cassette (ABC) transporter in *Phaeodactylum tricornutum* (Allen et al. 2008). To enhance iron uptake, cells also excrete strong organic ligands, reduce their cell size and requirement for iron (see Shaked and Lis 2012; Hassler et al. 2012 for recent reviews). All of these can result in the increased iron uptake rate under iron-limited conditions observed here (Fig. 3e, f). Please note that these experiments were conducted at relatively low growth irradiances (30 μmol photons m^−2^ s^−1^). The low light likely influenced the balance between the uptake of carbon and iron, as iron requirements tend to be higher at lower irradiances (Raven 1990). At higher light, for example, the lower electron transport and concomitant-increased photoprotective capacity in the +DFB cultures would have less of an impact on the uptake of iron than under low-light conditions.

The decline in iron incorporation in the presence of Van (Fig. 3e, f) suggests that iron uptake involves a high-affinity active transport system that is directly dependent on ATP (Allnutt and Bonner 1987). Whether this transport pathway is the commonly reported reductive pathway or a separate uptake pathway remains unclear. The stronger inhibition of iron uptake by Van in the +DFB treatment demonstrates an induction of this P-type ATPase transporter under iron limitation (Fig. 3f).

In accordance with previous studies, iron uptake by diatoms was significantly lower (60–90 %) in the presence of Asc, indicating that Fe(II) cannot be directly transported into the diatom, but that re-oxidation of Fe is required before it can be taken up (Maldonado et al. 2006). Functional studies and analysis of gene sequences further supported the existence of this re-oxidation step, which involves other essential redox trace metals such as Cu (Armbrust et al. 2004; Maldonado et al. 2006). The stronger effect of Asc in the +DFB treatment suggests that a surface reductase, followed by a re-oxidation step, is also used under iron limitation. The addition of ferrozine is sought to compete with the biological transport site for Fe(II) binding prior to re-oxidation and transport inside the cell (Shaked et al. 2005). However, iron uptake was not affected by Fer (Fig. 3e, f), which could suggest that Fer was not efficient in competing with Fe(II) cellular binding or that a Fe(III) transporter was present.

The strong electron acceptor MV, which provides uninterrupted linear electron transport and ATP production (Brooks et al. 1988), elicited a different response in the +Fe and +DFB treatments (Fig. 3e, f). In the +Fe cultures, there was a decline in Fe uptake, while it increased in the +DFB treatment. This could be explained by reductive transport pathways being different from the ATP-dependent uptake pathway, where iron-replete diatoms more heavily rely on the two-step redox reactions on the cell surface prior to uptake (Shaked et al. 2005), and iron-limited cells depend more on the P-type ATPase transport system for iron acquisition. The decline in iron acquisition in +Fe cultures in the presence of MV could then be due to the strong electron acceptor reacting with the surface reductase, blocking the re-oxidation step that is central for iron uptake in iron-replete cells. However, our data are yet insufficient to clearly demonstrate two distinct iron uptake pathways in this Antarctic diatom.

The addition of DCMU strongly reduced both the *F*_V_/*F*_M_ and ^14^C incorporation in +Fe and +DFB cultures (Fig. 3a–d). However, contrary to our expectation, DCMU had no effect on Fe uptake (Fig. 3e, f), suggesting that iron uptake can run on mitochondrial respiration alone. A similar observation was made in *P. antarctica* (Hassler, unpublished data). Carbon incorporation was strongly inhibited by CCCP, and although Fe uptake in presence of CCCP was not measured here, other studies have demonstrated that the Fe uptake by the algae *Chlorella* (Allnutt and Bonner 1987) was also inhibited in the presence of CCCP. This suggests that both C and Fe incorporation is dependent on the energy (ATP) generated from the transmembrane pH gradient.

## Conclusion

This study has investigated photosynthesis, iron and carbon uptake in the iron-limited Antarctic diatom *C. simplex*. There was a shift away from optimizing photochemistry toward enhancing photoprotection as indicted by the decline in photon absorption, the reorganization of energy partitioning through light-harvesting complexes and the increase in the relative proportion of diadinoxanthin. These physiological responses can be explained by a strongly reduced electron transfer capacity, where a lack of iron led to a reduction in electron transfer and subsequent partial inhibition of the photosystem. Being dependent on electron transport capacity, carbon fixation was strongly reduced under iron-limited growth conditions, which led to changes in iron acquisition strategy in *C. simplex*, inducing high-affinity transport pathways to maximize iron uptake. The observed changes in the photosynthetic processes as well as in carbon and iron uptake under iron limitation serve to highlight the strong influence iron can have on photochemistry and phytoplankton cell physiology.
